# Ambulance Problems Solved and Unsolved

**Published:** 1914-03-07

**Authors:** 


### The Hospital

The Workers' Newspaper of Administrative Medicine and Institutional Life, Administration, National Insurance and Health.

No. 1445, Vol. LV. SATURDAY, MARCH 7, 1914. PRICE ONE PENNY.

### AMBULANCE PROBLEMS SOLVED AND UNSOLVED.

After years of delay and the discussion of unworkable schemes and cheeseparing alternatives, it is at last fairly certain that London is to have a proper ambulance service provided by the one local authority which alone can usefully undertake it, the London County Council. A short time ago plans were in the air for the provision of an ambulance service by the Metropolitan Asylums Board, for which the County Council was to pay. We discussed the outlines of this scheme at the time (The Hospital, Dec. 6, 1913, p. 240); but it was speedily dropped when it became evident that the expense would be prohibitive. Now it is authoritatively announced that a new scheme has been practically completed, that it will be presented to the Council early this month, and that it follows substantially the lines suggested years ago by Sir William Collins. It must be a great source of gratification to Sir William to be thus vindicated after so many years of opposition. We congratulate him cordially on his coming triumph, and ourselves upon the privilege of having supported him and his views on this question.

From the announcements made public we gather that there will be seven or eight stations, at centres such as Lambeth, Fulham, and Hackney, and a system of alarm posts. The best points of the system which is in working operation in the City will be embodied, and the cost is estimated at approximately nine thousand pounds a year. If the cost really does not exceed this very moderate sum, and if the service is really as efficient as Sir William Collins and everyone else must wish to see it, London will have made a good bargain: such a good one, indeed, that the long years of waiting seem all the more unnecessary and regrettable. No estimates of the capital expenditure on stations, vans, motors, horses, buildings, and so forth have so far been published; so that an exact comparison with the recently deceased scheme for a service provided through the Metropolitan Asylums Board is not yet possible. But at least the cost of maintenance is very substantially less under this new scheme. It is, in fact, so cheap that the details will be keenly scrutinised for evidence as to the probable efficiency of the service. It is permissible to believe that no ambulance service is likely to be sanctioned by the London County Council which is not of the best; and there, for the present, the matter must be left until the plans are fully made public.

Turning to a broader aspect of ambulance work, we note that at a St. John Ambulance Brigade dinner in his constituency at Ilkeston the Minister for War stated that the King has agreed to the appointment of a committee, whose object is to effect a fusion between the Brigade and the British Red Cross Society. Some years ago, when the latter was an almost new organisation and was undertaking responsibilities under Mr. (now Lord) Haldane's Territorial Army Scheme which the St. John Association declined to shoulder, we pointed out in these columns that the Red Cross Society was taking what might prove to be a rash step from the financial standpoint. Whether the risks which it then decided to run have or have not materialised we do not know; nor is it necessary to inquire to what extent the St. John Association has been affected by the enterprise of the junior Society.

It is, however, obvious that the two bodies are doing almost identical work, and that it is wasteful and foolish thus to duplicate the machinery of organisation. It is quite clear that the St. John Association, which is venerable in point of age compared with the Red Cross Society and has a vastly greater prestige, cannot allow itself to be merged in the younger Society. And it is equally certain that the War Minister, who (together with his predecessor) owes a good deal to the British Red Cross Society's ardour and energy in connection with the Territorial Medical Service, would be most unwilling to see the Society come to grief. Whether it will prove to be possible to arrange terms of fusion which will be satisfactory to both agencies remains to be seen. Some such amalgamation is undoubtedly a common-sense business proposition. In fact, there should never have been allowed to come into existence two separate bodies having such similarity of aims. Unfortunately that cannot now be undone, and the "vested interests" which have sprung up in the two Societies may prove to be one of the many obstacles to any proposals for fusion.

A hint has been thrown out to the effect that the result of an inquiry, such as that foreshadowed by Colonel Seely, may be the partition of the country between the two contestants, by allotting to each a well-defined area for independent work. This suggestion seems to us unpractical and unstatesmanlike; it merely accentuates cleavage instead of abolishing it, and it does not get over the waste of money and energy involved by two organisations existing where one would suffice. It would get over the difficulty about finance, for presumably each Society would continue to raise its own funds as it might think best; but the membership would almost certainly suffer, since a good many workers might object to being handed over, as it were, to a rival Society, wherein their connection and reputation would be all to build up over again. We do not feel sanguine that this particular suggestion will prove to be feasible.

